# The Effects of Social Comparison and Subjective Financial Well-Being on Subjective Well-Being

**DOI:** 10.5964/ejop.14791

**Published:** 2025-08-29

**Authors:** Theda Renanita, Cicilia Larasati Rembulan

**Affiliations:** 1 Faculty of Psychology, Universitas Sebelas Maret, Surakarta, Indonesia; 2 Faculty of Psychology, Universitas Ciputra, Surabaya, Indonesia; University of Bari Aldo Moro, Bari, Italy

**Keywords:** social comparison, subjective financial well-being, subjective well-being, life satisfaction, happiness, Indonesia, IFLS

## Abstract

Existing research on the antecedents of subjective well-being (SWB), which comprises life satisfaction and happiness, remains inconclusive. Some studies suggest that increasing income enhances SWB, while others emphasize the role of income comparison within reference groups in influencing SWB. The role of subjective financial well-being in enhancing subjective well-being is also inconclusive. On the one hand, financial well-being may contribute to increasing SWB; on the other, subjective financial well-being is a characteristic compared to reference groups, and this comparison is what influences SWB. This study, therefore, sets out to answer the question: How does social comparison and subjective financial well-being influence SWB? We predict that social comparison influences SWB (life satisfaction and happiness) through the mediation of subjective financial well-being. Data from 3,591 respondents (1,750 females, 1,841 males, aged 21 – 60 years; *M* = 30.81, *SD* = 7.51) in the Indonesian Family Life Survey (IFLS 5) were analyzed using path analysis. The measures in this study include financial well-being, life satisfaction, happiness, and social comparison taken from IFLS-5 Book III A. The research findings indicate that social comparison does not directly influence life satisfaction and happiness. However, the influence of social comparison on both is mediated by subjective financial well-being. The practical implications of this research suggest that individuals who are happy and satisfied with their lives need to have at least one satisfying life domain, such as financial well-being, and focus on subjective self-evaluation rather than constantly comparing themselves with reference groups.

## Social Comparison and Well-Being

Both life satisfaction and happiness are fundamental components of subjective well-being. Happiness refers to a positive emotional state that typically arises when an individual’s social needs are met, such as feeling respected. Happiness encompasses both positive momentary positive affect and sustained emotional states such as contentment (Diener, Heintzelman et al., 2017). In addition, life satisfaction is more associated with holistic evaluations of life, including the fulfillment of basic needs such as food and shelter (Diener, Oishi & Tay, 2018).

Diener and Seligman (2018) emphasize the intrinsic aspects of subjective well-being, focusing on internal evaluations of life satisfaction and happiness. Studies supporting the intrinsic nature of well-being include Diener and Seligman (2018), who explain minimal differences in subjective well-being between affluent and developing countries. This suggests that wealth or money does not necessarily engender happiness and contentment. In accordance with this, Yates’ (2020) research supports the view that external factors, such as money, do not inherently enhance happiness. Nevertheless, financial management, philanthropy, and social connections can contribute to an individual’s happiness. Biswas-Diener (2008) further corroborates these findings concluding that external material luxuries do not significantly contribute to happiness or life satisfaction. As long as individuals can meet their basic needs, they do not require external, material luxuries.

Well-being has been extensively researched across various interdisciplinary fields, including psychology, economics, sociology, physiology, gerontology, and health sciences. Therefore, numerous definitions and terms are used to elucidate well-being, such as psychological well-being, quality of life, life satisfaction, domain satisfaction, and hedonic well-being. This research adopts Diener’s theory of subjective well-being due to its comprehensive and well-tested nature. Subjective well-being differs from eudaimonic well-being, which emphasizes psychological characteristics such as meaning in life, life purpose, positive social relationships, and autonomy (Diener, Lucas & Oishi, 2018). Diener’s perspective on subjective well-being, comprising cognitive and affective facets, aligns with studies by Frey (2017), Mureșan et al. (2021), and Staubli et al. (2014), which prioritize an individual's personal evaluation of their life over objective measures. Studying subjective well-being is crucial, as scholars have found it enhances health and longevity, fosters positive social relationships, improves job performance, and stimulates creativity (Diener, Lucas & Oishi, 2018; Diener, Pressman et al., 2017).

Subjective well-being does not solely center on internal facets. Some findings emphasize self-evaluation based on external conditions. Research indicates that individuals perceive happiness when their income surpasses that of others. Malka and Chatman (2003) illustrate that intrinsically driven individuals tend to report lower levels of happiness in their lives and have lower incomes. Hurka (2016) explains this phenomenon, suggesting that society promotes objectives considered ‘worth pursuing’, such as wealth, knowledge, achievement, and education. Individuals who lack these attributes may struggle to validate their relevance and functionality within society. The presence of others for comparison is crucial for individuals to feel happy and satisfied with their lives.

Wang et al. (2023) explain that an individual’s subjective well-being (SWB) is influenced by their perceived standing relative to others. Consistent with this notion, Ma and Piao (2019) indicates that social comparison among married Japanese career women can diminish their happiness. Carrieri (2012) discovered a similar phenomenon, indicating that social comparison significantly impacts subjective well-being, particularly when a gap exists between oneself and others. The contrast between inward-oriented and outward-oriented evaluations of well-being remains a subject of debate. Further research is needed to determine whether individuals experience greater happiness and life satisfaction by evaluating themselves or by comparing themselves to a reference group in their surroundings.

Individuals tend to strive for a deeper understanding of themselves, particularly within the context of social comparison. In seeking self-understanding, individuals often compare themselves with reference groups (Buunk & Gibbons, 2000; Chatterjee et al., 2019), particularly relying on external standards during uncertain situations (Forsyth, 2000). The tendency for social comparison is pronounced in younger individuals but declines as they approach the age of 60 (Buunk et al., 2020). Reference groups are individuals or groups whose attributes serve as benchmarks for comparison (van Praag, 2010). Common examples of reference groups include coworkers, close friends, and neighbors. Williams (1970) and Zell and Alicke (2020) highlight family as a significant reference group for most individuals.

Social comparison occurs in two directions: upward and downward. Upward comparison occurs when individuals compare their abilities, resources, or skills to a superior reference group. In contrast, downward comparison occurs when individuals compare themselves to those with inferior skills, resources, or abilities. Individuals generally prefer upward comparison when the opportunity arises, particularly in aspirational contexts. However in threatened situations, individuals may evaluate themselves differently, focusing on their standing relative to the reference group. When individuals perceive themselves as superior, they feel a sense of superiority; conversely, perceiving inferiority leads to feelings of inadequacy (Wheeler & Suls, 2020).

Upward comparison is often associated with negative emotions such as envy, guilt, regret, defensiveness, decreased subjective well-being (Buunk & Gibbons, 2000; White et al., 2006). These feelings arise when individuals perceive themselves as failing to achieve at the level of their reference group. Carraturo et al. (2023) elucidate that upward comparison can serve as an antecedent to depression. On the other hand, upward comparison can also have beneficial effects by motivating individuals to strive for greater achievements (Olivos et al., 2021). Downward comparison can provide a sense of relative prosperity, particularly for individuals experiencing psychological distress. Downward comparisons can also bring relief when individuals observe others in worse circumstances (Olivos et al., 2021). However, downward comparison is not recommended for long-term use as individuals may lose focus on effective problem-solving (VanderZee et al., 1996). Cheung and Lucas (2020) challenge the dichotomy, offering a balanced view of the effects of upward and downward comparisons. They argue that both upward and downward comparisons can positively and negatively impact well-being.

Social comparison is frequently examined through the social status of respondent’s family members, such as parents and parent-in-law (Alderson & Katz-Gerro, 2016; McFarland et al., 2001). Family social standing serves as a critical reference point in many cultures (Goldstein, 2015), particularly in collectivist societies like Indonesia (Setyaningrum et al., 2022). Parents and parents-in-law serve as primary social benchmarks, providing a framework for evaluating one's own achievements, financial stability, and social success. In marital contexts, comparisons with parents-in-law gain significance as individuals often feel compelled to meet or surpass their standards to attain acceptance and respect. This comparison can influence self-esteem, life satisfaction, and relationship dynamics within the family, as individuals evaluate their social position relative to the standards set by the older generation.

Existing studies demonstrate that social comparison arises from the desire to evaluate oneself against objective external standards. This aspiration for self-improvement often leads individuals to engage in upward comparisons. Research indicates that social comparison contrasts with theories of subjective well-being, which emphasize personal and internal evaluations over external standards. While social comparison seeks objective external validation, subjective well-being relies on personal and subjective self-evaluations. Subjective well-being is measured by an individual’s self-assessment of life satisfaction and happiness rather than external factors. Thus, frequent social comparison may negatively impact subjective well-being, as it shifts focus from internal self-assessment to external standards. Conversely, subjective well-being improves when individuals focus inwardly and reflect on their life satisfaction and happiness.

## Subjective Financial Well-Being and Subjective Well-Being

In recent decades, financial well-being has garnered significant attention across research disciplines including economics and psychology (Sorgente & Lanz, 2017). Broadly, the literature identifies three conceptual approaches to understanding financial well-being: the objective, the subjective, and a combined approach. The objective approach assesses financial well-being based on measurable economic indicators such as income and consumption levels (Sabelhaus & Manchester, 1995). In contrast, the subjective approach focuses on individual’s perceptions and evaluations of their financial status (Brüggen et al., 2017; Sabri et al., 2012; Zyphur et al., 2015). Within this framework, two key components are typically highlighted: experiential aspects-pertaining to perceived financial conditions and evaluative aspects relating to cognitive and emotional appraisals of one’s financial experiences (Sorgente & Lanz, 2017). For instance, Sabri et al. (2012) conceptualize financial well-being as a state of financial soundness, contentment, and freedom from worry. Similarly, Zyphur et al. (2015) describe it as a general attitude toward one’s financial situation, encompassing satisfaction, perceived financial challenges, confidence in financial management and future financial expectations. The combined approach, which integrates both objective and subjective dimensions, has been proposed to capture a more comprehensive understanding of financial well-being (Fergusson et al., 1981; Fletcher & Lorenz, 1985; Henager & Wilmarth, 2018; Joo, 1998).

Brüggen et al. (2017) conceptualize financial well-being as the perceived capacity to sustain one’s preferred standard of living and financial independence, both in the present and the future. Achieving financial well-being requires not only technical skills in managing financial activities but also the capacity to anticipate financial disruptions and to strategically pursue long-term financial objectives. Accordingly, personal financial behavior plays a pivotal role in shaping financial well-being at the individual level (Singh & Malik, 2022). These insights underscore the significance of the subjective approach in capturing the psychological and behavioral dimensions of financial well-being, which may not be fully addressed through objective financial indicators alone.

While individual-level factors play a key role, recent academic studies have expanded the discussion to include broader socioeconomic influences on subjective well-being. A literature review conducted by Das et al. (2020) identifies socioeconomic status particularly household income as a significant determinant of subjective well-being. In line with this, Brown and Gray (2016) highlight the household’s financial status as a key determinant of overall well-being. They further suggest that interhousehold financial comparisons can produce both detrimental comparative effects, potentially lowering satisfaction, and beneficial informational effects that may encourage constructive financial behavior. Furthermore, financial well-being is recognized as a core contributor to subjective well-being (Brüggen et al., 2017). Variations or disruptions in financial well-being have been linked to negative psychological and behavioral consequences, such as increased anxiety and declines in health conditions.

## Social Comparison, Subjective Financial Well-Being, and Subjective Well-Being

In economic studies, subjective well-being is associated with higher consumption levels and is related to personal utility (Stutzer & Frey, 2004). Meanwhile, Easterlin’s (1995) research shows that increased income does not increase welfare. However, income comparison between individuals influences well-being. When an individual perceives their income as lower than that of their reference groups, their subjective well-being tends to decline. Easterlin posits that an individual’s subjective well-being is positively related with personal income but negatively influenced by the average income of others. Accordingly, well-being is considered as a function of both absolute income and relative income in comparison to a reference group.

Extending this understanding, the present study examines the role of social comparison in shaping subjective well-being, operationalized as life satisfaction and happiness. Psychological literature increasingly acknowledges social comparison as a key mechanism influencing financial and emotional well-being. For example, Ferrer-i-Carbonell (2005) found that individuals’ happiness is significantly influenced by how their income compares to that of a reference group. Similarly, Alderson and Katz-Gerro (2016) observed that individuals who place greater emphasis on their relative income often report higher happiness levels although, paradoxically, those who view income comparison as essential may also experience lower well-being. These findings suggest that the psychological impact of social comparison is nuanced, shaped not only by relative income itself but also by the importance individuals assign to such comparisons.

A growing body of research has demonstrated the significant impact of social comparison on subjective well-being. For instance, Peck and Merighi (2007) found that comparisons related to health status significantly influence life satisfaction. Similarly, Bárcena-Martín et al. (2017) reported that upward comparisons where individuals compare themselves to those perceived as better off are generally associated with reduced subjective well-being, whereas downward comparisons tend to enhance it. These findings are supported by Olivos et al. (2021), who found that social comparison influences life satisfaction, with downward comparison positively affecting life satisfaction and upward comparison negatively affecting it. Dumludag et al. (2016) further emphasize that unmet expectations, often shaped by social comparisons, have a stronger negative influence on life satisfaction than expectations that are fulfilled. Underpinning these findings, Corcoran et al. (2011) explain that social comparison serves as a key mechanism through which individuals evaluate themselves relative to others.

Financial well-being represents another crucial determinant of subjective well-being. It encompasses individual’s evaluations of their current financial situation and their expectations regarding future financial stability (Riitsalu & van Raaij, 2022). Positive financial conditions have been linked to enhancements in various domains of well-being, including quality of life, happiness, mental health, quality of interpersonal relationships, and subjective and psychological well-being (Iannello et al., 2021; Nanda & Banerjee, 2021; Sorgente & Lanz, 2017; Spuhlera & Dew, 2019). Conversely, imbalances in financial well-being may contribute to diminished subjective well-being. For example, Downing (2016) found that financial difficulties are associated with negative psychological and behavioral outcomes, such as heightened anxiety, aggressive behavior, and deteriorating health. Similarly, Ryu and Fan (2023) identified that financial concerns ranging from worries about standard of living, medical expenses, and housing payments to health insurance, pensions, and monthly bills substantially increase psychological distress.

A systematic literature review by Bashir and Qureshi (2023) identifies several relative income attributes including relative income size, reference group income, relative income position, and income inequality as key antecedents of financial well-being. Palomäki (2017) supports this by demonstrating that retirees living in countries with higher average retirement incomes often perceive their own income adequacy more negatively than those in countries with lower average retiree incomes. These findings underscore the importance of relative evaluations, rather than absolute income, in shaping individuals’ financial satisfaction.

Expanding on this perspective, Neman (2020) suggests that income inequality affects financial well-being through its influence on financial behavior. As income inequality increases, individuals are more likely to allocate a larger portion of their income toward conspicuous consumption, often to purchase goods associated with social status. This pattern of expenditure, driven by social comparison, may ultimately undermine financial well-being. Supporting this view, Kim et al. (2017) report that individuals who perceive themselves as relatively deprived tend to prioritize materialistic values, which can exacerbate dissatisfaction with personal financial circumstances.

## Aim and Hypothesis of The Study

Numerous studies have examined the association between income and well-being (Diener & Seligman, 2018), as well as the effect of satiation on subjective well-being (Jebb et al., 2018; Stevenson & Wolfers, 2013). However, the mechanisms through which social comparison factors affect well-being particularly in the Indonesian context remain underexplored and conceptually underdeveloped. While prior research has addressed the role of social comparison in various populations, such as employees (Ferrer-i Carbonell, 2005), urban communities (Chatterjee et al., 2019), patients (VanderZee et al., 1996), and national contexts (Cheung & Lucas, 2020), the findings remain mixed. For example, social comparison did not significantly influence well-being in employee or urban settings, but it did in patient populations. In macro contexts such as countries, the financial position of a nation can influence well-being with outcomes potentially being either positive or negative. Social comparisons with countries with lower financial positions can result in feelings of hopelessness, relief, or pride. On the one hand, social comparison with countries with lower financial positions (downward comparison) may foster hope for greater prosperity. However, it can also diminish well-being by engendering a sense of being in a similar situation (Cheung & Lucas, 2020).

**Figure 1:**
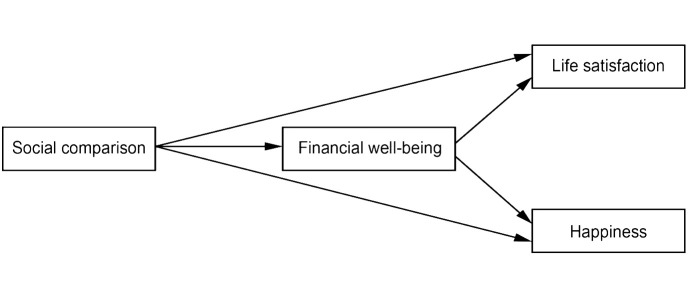
Model of the Influence of Social Comparison on Life Satisfaction and Happiness

These inconsistencies highlight a critical gap in the literature. To address this, the present study investigates the role of social comparison in shaping subjective well-being within the context of the Indonesian household. Unlike prior research focused on specific demographic or occupational groups, this study adopts a broader household-based approach, encompassing individuals of couples with or without children, in accordance with the United Nations’ (2020) definition of a household. The primary objective is to clarify the role of social comparison in well-being outcomes and to address these limitations and contradictions observed in existing literature. The model is illustrated in Figure 1.

Although previous research has examined the influence of social comparison and financial well-being on subjective well-being, the interrelationship among these three constructs remains insufficiently defined. This study aims to bridge that gap by examining whether subjective financial well-being serves as a mediating mechanism between social comparison and subjective well-being.

Specifically, this study investigates the impact of social comparison on subjective well-being, specifically examining its influence on two key dimensions: life satisfaction and happiness. Additionally, this study explores the mediating role of subjective financial well-being in these relationships.

**Research Hypotheses**
**H1:** Subjective financial well-being mediates the relationship between social comparison and life satisfaction.
**H2:** Subjective financial well-being mediates the relationship between social comparison and happiness.

## Method

### Participants

The data used in this study were retrieved from the Indonesian Family Life Survey (IFLS-5), conducted by the RAND Corporation. IFLS is a collaboration between RAND Corp and Survey Meter. IFLS is a longitudinal survey focuses on the health and socioeconomics of approximately 80,000 individuals and 10,000 households across 13 provinces in Indonesia. The IFLS surveys and procedures received thorough review and approval from Institutional Review Boards (IRBs) in both the United States at RAND, and in Indonesia at Gadjah Mada University (UGM).

The data were collected from 2014 to 2015 (Strauss et al., 2016). This study uses data on financial well-being, life satisfaction, happiness, and social comparison among the available variables. The IFLS comprises multiple books; however, the variables relevant to this study are located specifically in Book IIIA. The data analyzed is data from 2014 – 2015, where the information related to subjective well-being was available. The variables studied are not affected by time, therefore this data still provides information on the influence between variables.

Participants were selected based on several inclusion criteria. The criteria encompassed being aged between 21 and 60, married marital status, and completion of the financial well-being, life satisfaction, happiness, and social comparison scales. Exclusion criteria involved responses indicating uncertainty or non-responsiveness in the financial well-being scale, including responses such as “Dead”, “Didn't want to answer”, and “Don't know”. Based on these criteria, 3,591 participants were eligible for this study. The participants consisted of 1,750 (48.7%) women and 1,841 (51.3%) men, with a *M*_age_ of 30.81 (*SD* = 7.51).

### Measurement

#### Financial Well-Being

In this study, financial well-being is measured by a three-factor model encompassing three aspects, which are subjective wealth (SW01–SW02), the perceived future standard of living (SW03 – SW03a), and current standard of living (SW03b–SW04) (Ghina & Sukarno, 2021). The model comprises six items, all sourced from IFLS-5 Book IIIA. The author first checked the fit model using several fit indices. The model fit indices used are Chi-square, Root Mean Square Error of Approximation (RMSEA), Comparative Fit Index (CFI), Tucker Lewis Index (TLI), and Standardized Root Mean Square Residual (SRMR). Ideally, Chi-square is not significant (*p* > .05), RMSEA < .08, CFI/TLI > .95, and SRMR < .08 (Wang & Wang, 2020). This model fits the data, which is supported by the results of confirmatory factor analysis (χ^2^ (6) = 150.470, *p* < .001, RMSEA = .082 [.071, .093], SRMR = .040, CFI = .962, TLI = .906), with factor loadings ranging from .337 to .937.

#### Life Satisfaction

Life satisfaction is assessed using a single question (SW00) sourced from IFLS-5 Book IIIA, which prompts participation to evaluate their overall life satisfaction. The question reads: “Please think about your life as a whole. How satisfied are you with it?” Respondents provide their level of satisfaction using a five-point scale, namely (1) *Completely satisfied*, (2) *Very satisfied*, (3) *Somewhat satisfied*, (4) *Not very satisfied*, and (5) *Not at all satisfied*. Then, researchers reversed the scores so that a larger scale indicated higher life satisfaction.

#### Happiness

Happiness is measured by a single question (SW12) in IFLS-5 Book IIIA, which prompts, “Taken all things together, how would you say things are these days? Would you say you were very happy, happy, unhappy or very unhappy?” These items were taken from the United States General Social Survey (Strauss et al., 2016).

#### Social Comparison

Social comparison was assessed using questions that compared the social status of the participant's parents with that of their in-laws. The questions were sourced from IFLS-5 Book IIIA and used by Ghina and Sukarno (2021). The main question is, “At the time that you were married/first cohabitant, how did the status of your parents compare to the status of your parents-in-law?” (PK21A – PK21H). The status in question is educational and employment status and financial status. The educational and employment status compared were the father's occupation, father's education, and mother's education. Financial status includes position in society, housing quality, income, land ownership, and non-land assets. There are five response options ranging from (1) “High” to (5) “Low”. The author reverses the scores so that a larger scale indicates the status of the mother/father (parents) is higher than that of the parents-in-law. The author first checked the fit model using several fit indices. The model fit indices used are Chi-square, root mean square error of approximation (RMSEA), Comparative Fit Index (CFI), Tucker Lewis Index (TLI), and Standardized Root Mean Square Residual (SRMR). Ideally, Chi-square is not significant (*p* > .05), RMSEA < .08, CFI/TLI > .95, and SRMR < .08 (Wang & Wang, 2020). Confirmatory factor analysis results supported the validity of this model (χ^2^ (17) = 88.246, *p* < .001, RMSEA = .034 [.027, .041], SRMR = .024, CFI = .983, TLI = .971).

### Data Analysis

#### Descriptive Analysis

The results of the descriptive analyses are presented in Table 1. The participants consisted of 1,750 (48.7%) women and 1,841 (51.3%) men. The age range of the participants was 21 – 60 years with a *M*_age_ of 30.81 (*SD* = 7.51).

**Table 1:** Descriptive Statistics

Variable	*N*	%
Gender		
Male	1,750	48.7
Female	1,841	51.3
Education		
Elementary school	679	18.9
Junior high school	783	21.8
Senior high school	1371	38.2
University	687	19.1
Postgraduate	28	0.8
Missing	43	1.2

#### Path Analysis

The model tested involved the variables *Life satisfaction* and *Happiness* as dependent variables, *Financial wellbeing* as a mediator and *Social comparison* as an independent variable. The author conducted model testing using path analysis to predict happiness and life satisfaction by testing theoretical and logical relationships based on correlations between variables (Joo & Grable, 2004; Wang & Wang, 2020). The analyzed data consists of factor scores, which are claimed to be more accurate than other scores (Grice, 2001). The estimator used is maximum likelihood and 10,000 bootstrap samples. The alpha level (*p*) for determining statistical significance was set at .05. All analyses were conducted using Mplus 8.3 (Muthén & Muthén, 2017).

## Results

The results of the intercorrelation analysis between variables show that most variables show significant correlations. However, the social comparison variable does not correlate significantly with subjective well-being indicators such as life satisfaction and happiness. Table 2 presents the means, standard deviations, and intercorrelations between the variables.

**Table 2:** Mean, Standard Deviation, and Intercorrelation Between Variables

Variable	*M*	*D*	1	2	3	4
1. Financial well-being	-.002	.850	1			
2. Life satisfaction	3.380	.785	.353**	1		
3. Happiness	3.143	.469	.298**	.324**	1	
4. Social comparison	-.001	.887	-.078**	-.030	-.013	1

*p < .05. **p < .01.

**Figure 2:**
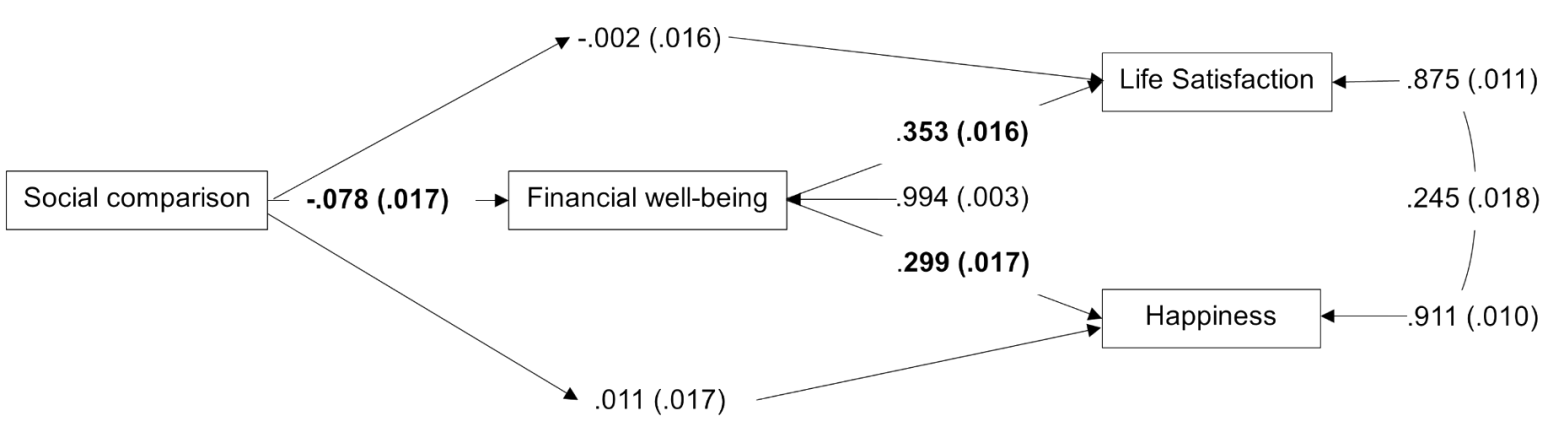
Path Analysis Results (Standardized Coefficients) *Note*. Bold text indicates significant relationships

The analysis indicates that the direct effect of social comparison on life satisfaction is insignificant (β = -.002, SE = .016, *p* = .886). However, subjective financial well-being significantly influences life satisfaction (β = .353, SE = .016, *p* < .001). The analysis results indicate a significant effect of social comparison on subjective financial well-being (β = -.078, SE = .017, *p* < .001). Furthermore, the analysis found that subjective financial well-being mediates the influence of social comparison on satisfaction (β = -.027, 95% CI [-.040, -.016]). These results supported the proposed Hypothesis 1. (See Figure 2).

The direct effect of social comparison on happiness was not significant (β = .011, SE = .017, *p* = .538). Additionally, the analysis revealed that subjective financial well-being significantly impacts happiness (β = .299, SE = .017, *p* < .001). Additionally, subjective financial well-being mediates the influence of social comparison on happiness (β = -.023, 95% CI [-.034, -.013]). These results supported the proposed Hypothesis 2. The unstandardized path analysis can be found in Table 3.

**Table 3:** Unstandardized Path Analysis for Each Variable

						95% CI	
Effect	β	B	SE	z-value	*p*	LL	UL
Indirect							
SC → FWB → LS	-.027	-.024	.005	-4.539	< .001	-.035	-.014
SC → FWB → H	-.023	-.012	.003	-4.393	< .001	-.018	-.007
Direct							
SC → FWB	-.078	-.074	.016	-4.658	< .001	-.106	-.043
FWB → LS	.353	.326	.015	21.567	< .001	.296	.356
FWB → H	.299	.165	.011	15.516	< .001	.144	.186
SC → LS	-.002	-.002	.014	-0.144	.886	-.028	.026
SC → H	.011	.006	.009	0.616	.538	-.013	.024

*Note.* CI = Confidence Interval, LL = Low Limit, UL = Upper Limit, SE = Standard Error, SC = Social comparison, FWB = Financial Well-Being, LS = Life Satisfaction, H = Happiness.

**p < .01.

## Discussion and Conclusion

This study examines how social comparison and financial well-being impact happiness and life satisfaction, which are key components of subjective well-being. The findings reveal that social comparison does not exert a significant direct effect on life satisfaction and happiness. This suggests that individuals do not achieve subjective well-being by comparing themselves with their reference group. These results diverge from those of Dumludag et al. (2016) and Olivos et al. (2021), who found that social comparison reduces life satisfaction, one of the components of subjective well-being. Olivos explains that social comparison can reduce life satisfaction as it often leads to feelings of envy rather than eliciting emotions of gratitude. In contrast, life satisfaction is more likely to arise when individuals experience feelings of gratitude towards their lives. Our research findings indicate that social comparison does not negatively affect life satisfaction and happiness may be due to the different measurement tools used. Dumludag et al. (2016) and Olivos et al. (2021) distinguish between upward and downward comparisons in their studies, whereas this research does not differentiate between upward and downward comparisons. This measurement tool used in this study focused on comparing the status between participants and their reference group. It would be beneficial for future researchers to use a more detailed measurement tool that distinguishes between upward and downward comparisons. The use of a generic social comparison measure in this study may be one of its limitations.

The relatively low *R*^2^ value of 0.005 for the effect of social comparison on financial well-being indicates that, although the effect is significant, social comparison explains only a small portion of the variance in financial well-being. First, the reference group used in this study is family. In Indonesia, it is quite common for family members to lend money to one another, particularly in urgent situations such as covering educational expenses for children or during times of illness. It is likely that family does not serve solely as a reference group that only evokes envy but may instead function as a source of social support. For future researchers using social comparison variables, a more nuanced consideration of participant-relevant reference groups is recommended. Second, other factors, such as decision-making, financial literacy, personality, and lifestyle, may also play a role. Future researchers could consider these additional variables, as their exclusion represents a limitation of this study.

The analysis highlights that financial well-being significantly impacts happiness and life satisfaction, with its influence on life satisfaction being greater than on happiness. These findings corroborate previous research indicating that financial well-being positively affects overall well-being (Brüggen et al., 2017; Riitsalu & van Raaij, 2022). Conversely, financial problems are associated with decreases in both happiness and life satisfaction.

An intriguing aspect of this study is the mediating role of financial well-being in the relationship between social comparison and subjective well-being. While social comparison alone does not directly affect subjective well-being, it can influence it through individuals’ evaluations of their financial conditions. Subjective financial well-being focuses on an individual’s perception of their financial status, independent of social comparison with reference groups. Subjective financial well-being centers on evaluating an individual’s financial condition in the present and future. This highlights the importance of the subjective-personal aspect oriented inwardly in evaluating specific life domains, such as finances, as well as overall life domains, such as happiness and life satisfaction. One can attain happiness and life satisfaction by integrating outward-oriented (social comparison) and inward-oriented (subjective financial well-being) orientations. Solely outward orientation cannot engender happiness and life satisfaction in a person's life.

This study indicates that life satisfaction and happiness are primarily inward, personal, and subjective. To achieve these states, individuals should engage in introspective assessment based on personal standards rather than reference group standards. Discrepancies between one's condition and that of a superior reference group can lead to life dissatisfaction and unhappiness. This aligns with Biswas-Diener (2008), Diener, Heintzelman et al. (2017), Ma and Piao (2019), and Yates (2020), who assert that happiness and life satisfaction are viewed from a personal perspective, contrary to Hurka’s (2016) emphasis on an individual’s functionality within society. This study also differs from Easterlin (1995), who emphasizes happiness measured through comparisons with others, as well as from Wang et al. (2023) and Malka and Chatman (2003), who focus more on happiness and life satisfaction from extrinsic measures.

In the Indonesian context, Nugroho et al. (2022) examined the influence of social capital on subjective well-being across rural and urban settings, without including financial well-being or social comparison as explanatory variables. Moreover, their emphasis on urban-rural differences contrasts with the present study’s focus on the household units as the level of analysis. This distinction highlights our study’s contribution in integrating social comparison and financial well-being as psychological mechanisms underlying subjective well-being, beyond structural context such as the urban-rural setting. Similarly, Sujarwoto (2021), using IFLS panel data, demonstrates that happiness positively predicts economic and social outcomes such as income, employment, health, and marriage. His study positions happiness as an antecedent of future financial conditions. However, neither social comparison nor subjective financial appraisals are considered in that model. Ghina and Sukarno (2021) focused on objective financial indicators and found that relative income negatively affects financial well-being, yet they did not examine its psychological consequences. In contrast, this study offers a psychological perspective by positioning subjective financial well-being as a mediator between social comparison and subjective well-being, thereby bridging external comparisons and internal evaluations to explain life satisfaction and happiness more comprehensively. This study supports a conceptualization on financial well-being that is grounded in psychological appraisal rather than solely in economic indicators.

We acknowledge the broad age range of 21 – 60 years used in this study may encompass significant personal, social, and professional developmental differences that can influence subjective well-being. This range was selected to capture the general adult population. However, we recognize that developmental differences within this range could impact subjective well-being. Therefore, we have included this as a limitation and suggest future research could explore narrower age ranges to capture these nuances more accurately.

These findings imply that while objective awareness through social comparison can be useful, individuals need at least one positive evaluation in a life domain, such as finances, to achieve subjective well-being (satisfaction and happiness). Over-reliance on social comparison cannot directly enhance life satisfaction and happiness.

### Future Research Implication

This research focused on subjective variables. Social comparison is a subjective variable oriented toward external conditions, namely the reference group. In a similar vein, subjective financial well-being reflects individuals’ internal assessments of their financial situation. The dependent variable, subjective well-being, consisting of happiness and life satisfaction is likewise based on self-evaluative judgements. To enhance the comprehensiveness of future research, combining objective indicators of well-being is recommended. Although these findings highlight the importance of subjective financial well-being, societal reality demands individuals to be relevant and contribute to their surroundings, often requiring more than internal financial evaluation alone. Individuals need to contribute to society, making the objective aspect of asset ownership and wealth increase essential and warrants further review. Objective well-being aspects, such as material possessions and quality of life (Alatartseva & Barysheva, 2015), can be further explored in future studies.

This research utilized big data from the IFLS. The measurement tool used to examine social comparison did not distinguish between upward and downward comparisons. Future research should distinguish between these two aspects of social comparison to provide a more nuanced understanding. Additionally, conducting qualitative research or developing relevant measurement tools for specific communities would be beneficial, as social comparison behavior, evaluations of financial well-being, and personal well-being are closely related to local culture (Muresan et al., 2020).

### Practical Implication

Based on the research findings, the following recommendations are proposed: a) reduce engagement in social comparison, b) enhance personal economic or financial capabilities, c) increase satisfaction with one’s financial abilities, d) establish personal standards for the well-being goals one aims to achieve.

## Supplementary Materials

**Table:** 

Type of supplementary materials	Availability/Access
Data	
Study data set.	Rembulan and Renanita (2025)

## Data Availability

Data can be accessed through the Open Science Framework. See Rembulan and Renanita (2025).
